# Randomised controlled trial of a 12 week yoga intervention on negative affective states, cardiovascular and cognitive function in post-cardiac rehabilitation patients

**DOI:** 10.1186/1472-6882-14-411

**Published:** 2014-10-24

**Authors:** Alan Yeung, Hosen Kiat, A Robert Denniss, Birinder S Cheema, Alan Bensoussan, Bianca Machliss, Ben Colagiuri, Dennis Chang

**Affiliations:** National Institute of Complementary Medicine, School of Science and Health, University of Western Sydney, Locked Bag 1797, Penrith, NSW 2751 Australia; Cardiac Health Institute, Eastwood, Australia; Departments of Cardiology, Westmead and Blacktown Hospitals, Sydney, Australia; Yoga Synergy, Sydney, Australia; School of Psychology, University of Sydney, Sydney, Australia

**Keywords:** Yoga, Anxiety, Stress, Depression, Cardiac rehabilitation

## Abstract

**Background:**

Negative affective states such as anxiety, depression and stress are significant risk factors for cardiovascular disease, particularly in cardiac and post-cardiac rehabilitation populations.

Yoga is a balanced practice of physical exercise, breathing control and meditation that can reduce psychosocial symptoms as well as improve cardiovascular and cognitive function. It has the potential to positively affect multiple disease pathways and may prove to be a practical adjunct to cardiac rehabilitation in further reducing cardiac risk factors as well as improving self-efficacy and post-cardiac rehabilitation adherence to healthy lifestyle behaviours.

**Method and design:**

This is a parallel arm, multi-centre, randomised controlled trial that will assess the outcomes of post- phase 2 cardiac rehabilitation patients assigned to a yoga intervention in comparison to a no-treatment wait-list control group. Participants randomised to the yoga group will engage in a 12 week yoga program comprising of two group based sessions and one self-administered home session each week. Group based sessions will be led by an experienced yoga instructor. This will involve teaching beginner students a hatha yoga sequence that incorporates asana (poses and postures), pranayama (breathing control) and meditation. The primary outcomes of this study are negative affective states of anxiety, depression and stress assessed using the Depression Anxiety Stress Scale. Secondary outcomes include measures of quality of life, and cardiovascular and cognitive function. The cardiovascular outcomes will include blood pressure, heart rate, heart rate variability, pulse wave velocity, carotid intima media thickness measurements, lipid/glucose profiles and C-reactive protein assays. Assessments will be conducted prior to (week 0), mid-way through (week 6) and following the intervention period (week 12) as well as at a four week follow-up (week 16).

**Discussion:**

This study will determine the effect of yoga practice on negative affective states, cardiovascular and cognitive function in post-phase 2 cardiac rehabilitation patients. The findings may provide evidence to incorporate yoga into standardised cardiac rehabilitation programs as a practical adjunct to improve the management of psychosocial symptoms associated with cardiovascular events in addition to improving patients’ cognitive and cardiovascular functions.

**Trial Registration:**

ACTRN12612000358842

## Background

Cardiovascular disease (CVD) is a leading cause of morbidity and mortality worldwide. In 2004, CVD caused 17.1 million deaths and this is estimated to increase to 23.6 million by 2030 [1]. This increase is paralleled by an increase in the prevalence of psychological risk factors such as anxiety, depression, and stress that have been shown to be associated with CVD morbidity and mortality [2, 3]. Anxiety and depression are common after CVD events with prevalence rates up to 31%. Interestingly, anxiety and depression in CVD patients appear to be independent of functional health, time between cardiac incidents, and enrolment in cardiac rehabilitation (CR) programs [4–6]. Furthermore, these psychosocial factors often cluster within an individual with a significant number of patients presenting with multiple psychological symptoms upon CR entry [5]. This evidence is particularly concerning as these affective symptoms have been shown to increase the risk of primary and subsequent cardiovascular events, and contribute to poor behavioural aspects that affect general cardiovascular health [7–9]. While anxiety and depression are distinctly separate at a conceptual level, past attempts to quantify these constructs yielded high degrees of inter-correlation [10]. This has resulted in the development of a tripartite continuum with anxiety and depression at opposite extremes and a centralised nonspecific component, which encompasses key aspects of stress [11].

Numerous studies have demonstrated that psychosocial stress is a significant risk factor for CVD in both patients with established disease and healthy individuals [7, 12] and large scale community-based studies as well as prospective studies have found significant relationships and prevalence rates between anxiety, depression and CVD risk [13–15]. Whilst the precise physiological and biochemical mechanisms that underlie the bidirectional associations between these negative affective states and CVD are not well understood, a number of studies have implicated hypothalamic-pituitary-adrenal axis dysfunction, activation of pro-inflammatory cytokines, autonomic dysregulation and increased markers of inflammation as possible key neurobiological mechanisms [16–18]. In addition to this, previous evidence indicates negative behavioural patterns in patients with high anxiety or depression such as less contact with their cardiologists, being less likely to seek preventative medical care, poorer medication compliance as well as reduced self-motivational, and physical inactivity that further worsens cardiac outcomes [5, 19]. Furthermore, patients with anxiety or depression, even at subclinical levels exhibit significantly lower quality of life (QoL) scores [5, 20].

In an attempt to reduce the prevalence and progression of CVD, cardiac rehabilitation and prevention programs which incorporate exercise regimens, stress management techniques and patient education, have gained widespread acceptance and have been shown to improve risk factor management and functional capacity thereby reducing the chance of secondary cardiovascular incidences [21]. CR programs have also been shown to improve cognitive function, QoL, and psychosocial outcomes and programs with an exercise component also have benefits on mortality. However in spite of these benefits, CR programs remain underutilised with only an estimated 30% of eligible patients in Australia participating [22, 23]. Whilst it is widely believed that continued exercise and physical activity is required to sustain most of the benefits gained through the participation of CR, studies have reported an adherence rate to physical activity of 30% to 60% in those who complete phase 2 rehabilitation programs [24, 25]. This has been highlighted in a study of myocardial infarction (MI) patients where CR benefits diminished to a level in which no significant differences between CR and non-CR participants could be seen 6 months post-MI [26]. Therefore, a practical adjunct which can improve physiological and psychosocial risk factors may further reduce cardiac risk factors and improve self-efficacy and long-term adherence to healthy behaviours.

Yoga may be one such treatment [27]. It is described as a path, which integrates the body, senses, mind, and intelligence with the self and numerous studies have reported the benefits of yoga in reducing stress, anxiety, and depressive symptoms in healthy and psychologically affected volunteers [28–30]. This is consistent with numerous reviews that highlight the positive potential of yoga for the treatment of depressive and anxiety disorders [31–34]. Whilst the methodological quality of prior yoga studies are generally weak, there are multiple reviews that highlight the favourable effects of yoga in improving the cardiovascular profile across a range of clinical and healthy populations [35–37]. Significant improvements in a wide range of cardiovascular parameters such as the high frequency component of heart rate variability (HRV), pulse wave velocity (PWV), blood lipid profiles (low density / high density lipoproteins and triglycerides), and C-reactive proteins (CRP) highlights the broad range of effect yoga has on the cardiovascular system [37–45]. Hatha yoga is consider to achieve benefits in blood circulation by enhance the effect of circulatory pumps in the body. Dynamic exercises such as *vinyasas* focus on activating and relaxing muscles during movement to enhance the effects of the musculoskeletal pump. Furthermore co-activation of antagonistic muscles during *vinayasas* and static postures (*asanas*) can create regions of relatively low and high pressures to augment muscle co-activation and postural circulation pumps to improve blood circulation [46].

To complement the array of psychosocial and cardiovascular impact of yoga, recent studies linking the detrimental effects of CVD and arterial stiffness with the decline of cognitive performance provides a framework for the investigation into the effects of yoga and its influence on cognitive function [47–49]. Whilst there are few studies which assess the effect of yoga on neuro-cognition, considering its capacity to improve a wide range of cardiovascular parameters, there may be corresponding improvements in the cognitive profile. Preliminary evidence in a study by Cohen et al. [50] demonstrates a strong association between the executive abilities of focused attention, processing speed, maintenance of effort, and QoL improvements after a yoga intervention in CR patients [50]. Whilst there is a significant body of evidence to support its use in healthy, psychosocially affected and various pathological populations, there are relatively few studies that examine a combination of psychosocial, cardiovascular, and cognitive effects in cardiac or CR populations. Given the impact of yoga across a broad range of health adaptations in various populations, we hypothesise that yoga will improve anxiety, depression and stress accompanied by improvements in cardiovascular parameters of HRV, PWV, CIMT, biomarkers and cognitive function in post-CR patients.

## Method and design

### Study design

This is a two arm, multi-centre, parallel-arm randomised controlled trial that will compare the outcomes of patients with cardiovascular disease assigned to a 12 week yoga intervention or to a no-treatment control group. The study will be conducted in approximately 134 post-CR participants over a period of 16 weeks comprising of a 12 week intervention period and 4 week follow up assessment. The primary outcome of this study is the component and overall scores obtained through the Depression Anxiety Stress Subscale (DASS) [51]. Secondary outcomes include QoL and cardiovascular and cognitive function. Timepoints for assessments will take place at baseline (week 0), mid-intervention (week 6), post-intervention (week 12), and at follow up (week 16). Ethics approval has been sought from the University of Western Sydney (H9402), Sydney Adventist Hospital Group (2011.40) and Western Sydney Local Health District Human Research Ethics Committees (2013/6/4.5(3747)).

### Sample size and power calculation

To our knowledge, there is at present only one study which examines the effect of yoga training in post-CR patients [52]. This study assessed the effects of a 6 week yoga program on lipid profiles and brachial artery vasodilation in 10 post-CR patients and 23 healthy individuals however published data was insufficient to conduct a preliminary power analysis [52]. Given the DASS is a primary outcome measure in this study, a power analysis was conducted upon the results published in a meta-analysis on yogic effects in depression [34]. Results published by Cramer et al. [34] noted a standardised mean difference of 0.69 compared to usual care across five studies. Application of these findings as a basis for an approximated power calculation resulted in an estimated required sample size of 112 participants. Allowing a 20% drop-out rate, a total of 134 patients will be recruited in the current study. This power calculation has been conducted based on a population other than post-cardiac rehabilitation due to a lack of available data in this patient cohort to permit a more robust calculation. As such, an interim sample size analysis will also be conducted at n = 50 allowing for an acceptable estimate of the population standard deviation and hence considerably more robust sample size calculation to ensure adequate power.

### Participants

Eligibility criteria includes: Adult (>18 years) having proven coronary artery disease and completion of a Phase 2 cardiac rehabilitation program within the past 9 months; available to attend two yoga sessions per week and complete a simplified self-administered yoga program once a week for the duration of the study; not currently or previous engaged in a yoga program (>3 yoga session); no acute or chronic medical conditions which would make yoga potentially hazardous; ability to communicate in English; willingness and cognitive ability to provide written informed consent. Should the participant have any of the following conditions they will be excluded from the trial: pacemakers or implanted defibrillators, pregnancy; end stage congestive heart failure; permanent bed-bound status; unstable abdominal, thoracic or cerebral aneurysm; acute myocarditis, pericarditis, pulmonary embolus or pulmonary infarction; severe cognitive deficits (MMSE < 21); previous or current psychological disorders that are not associated with depression or anxiety.

### Randomization

Participants will be randomized via computer-generated randomly permuted blocks into interventional group or control group. The randomization assignments will be prepared by a National Institute of Complementary Medicine (NICM) Research Program Coordinator who is external to the research team. Group assignment will be delivered to participants in sealed envelopes once the participant is screened and fully enrolled.

### Intervention

#### Experimental group

Participants randomised to the yoga therapy group will receive two supervised yoga sessions per week for a period of 12 weeks. They will also be provided with a simplified take-home yoga program, which they will be instructed to practice once per week. The yoga sessions will be group-based and led by an experienced yoga instructor from Yoga Synergy Pty Ltd (Sydney, Australia) [46]. These sessions will run twice per week for approximately one hour per session (details of yoga sequence and time requirements outlined in Table 1). The hatha yoga program will be similar to that used by the research team in a previous study [45] and will involve teaching beginner students safely and progressively over 12 weeks incorporating *asanas* (poses and postures), *pranayama* (breathing control) and *savasana* (meditation). At the start of each session, a series of introductory movements designed to warm-up the extremities, large joints and spine (*itkata vinyasa* and *utkata danda nadi vinyasa*) will be conducted before moving into a salute to the moon (*candra namaskar*). This will be followed by standing postures which are linked to one another to warm up the body whilst stretching and strengthening the muscles (*trikonasa*, *parsvakona*, *gadja hasta padottana*, *gadja baddha padottana*, *parsvottonasa*, *eka pada*, *virabhadra*). Following this, a series of floor postures which are also linked together will be conducted and include: forward bends, twists, hip opening, back arches, and back arch releases. As participants become more competent with training, an inversion (shoulderstand) is added to help calm and restore the body and nervous system. This is followed by a series of neck and spine releases to ensure participant safety. Participants then sat on the floor and engaged in breathing exercises (pranayama) and then supine meditation/relaxation (*savasana*). All postures and exercises are comprised of a simple and more challenging variant to allow difficulty to be scalable within the yoga program in relation to participant ability and capacity.

**Table 1:** **Yoga routine**

Introductory section	
5 Minutes	Utkata Vinyasa
	Utkata Danda Nadi Vinyasa
	Candra Namaskar
	Surya Namaskar
Standing Postures	
10 Minutes	Trikonasa Vinyasa
	Parsvakona Vinyasa
	Gadja Hasta Padottana Vinyasa
	Gadja Baddha Padottana Vinyasa
	Parsvottonasa Vinyasa
	Eka Pada Vinyasa
	Virabhadra Vinyasa
Floor Postures	
10 Minutes	Pashima Vinyasa
	Janu Sirsa Vinyasa
	Maricy Vinyasa
	Baddha Kona Vinyasa
	Pada Sirsa Preparation Vinyasa
Back Bending Sequence	
5 Minutes	Adho Viparita Vinyasa
	Adho Viparita Vinyasa
	Urdhava Dhanura Vinyasa
Back Bend Releasing Sequence	
5 Minutes	Urdhva Dhanura Releasing Vinyasa
	Hasta Vinyasa
Shoulderstand, Releasing Postures & Finishing Postures	
10 Minutes	Sarvangasana Vinyasa
	Viparita Mudra Vinyasa
	Padma Vinyasa
Pranayam Meditation & Relaxation	
15 Minutes	Nadi Padma Namaskar
	Nadi Sodhana Pranayama
	Savaasana

#### Control group

Participants randomised to the wait list control group will receive no instructions about yoga practice for the 16 week trial period (12 week intervention +4 week follow up). Upon their completion of the trial, wait list participants will be given the opportunity to participate within the yoga program.

### Outcomes

Participants will be restricted from consuming caffeine 4 hours prior to and alcohol 24 hours prior to each of the four testing sessions (baseline, mid-intervention, post-intervention, follow-up). This includes caffeinated products such as caffeine tablets (No-Doz, etc.) as well as caffeinated drinks.

### Psychological outcomes

#### Depression Anxiety Stress Scales (DASS)

The DASS is a set of three self-reported scales designed to assess the negative emotional states of depression, anxiety and stress. Each of the three DASS sub-scales contains 14 items, divided into subscales of 2–5 items with similar content. The DASS has been employed in a number of studies in cardiac populations and has been shown to have good test-retest reliability coefficients for all scales [53]. The DASS scale was found to show better discrimination when assessed in comparison to a well-established coronary prone behavioural pattern scale such as the Framingham Scale in a group of Myocardial Infarction (MI) and matched control patients [54]. While not as widely used as other questionnaires of anxiety and depression such as the State Trait Anxiety Inventory, Beck Depression Inventory or Beck Anxiety Inventory, the DASS shows greater discriminant validity in regards to these psychosocial factors [11]. Participants will be asked to use 4-point severity/frequency scales to rate the extent to which they have experienced each state over the past week. Overall DASS scores will be calculated and presented for statistical analysis.

#### Short Form 36 version 2 (SF36v2)

Short Form 36 is a psychometrically robust and clinically credible 36–item questionnaire that is designed to assess health related quality of life across diverse medical and psychiatric groups [55, 56]. It has also been shown to be a valid and reliable instrument for assessing physical and mental health status of cardiac patients and that CVD is associated with reductions in health-related QoL [57]. The SF36v2 organises the 36 items into eight components and presented on a percentage scale (0–100) and T-score. These eight components will be combined into two summary measures to provide overall estimates of physical and mental health. SF36v2 will be presented to participant as either a paper based questionnaire or as an electronic survey using Evado clinical trial software (Evado, Melbourne, Australia).

#### Cardiovascular measurements

Heart Rate and Blood Pressure: All blood pressure and heart rate measurements will be calculated using an automatic sphygmomanometer, designed for professional use (Omron, Sydney, Australia). Brachial arterial blood pressure will be taken following a 5 minute rest period. This will be done with an automatic sphygmomanometer whilst the participant is sitting comfortably.

Heart Rate Variability (HRV): HRV has been extensively used to assess vagal function and the associations between autonomic imbalance and disease morbidity and mortality [58]. To date, there is a large body of evidence indicating that HRV is an independent predictor of CVD morbidity and mortality in both high and low risk populations [59–62]. Whilst large population studies have highlighted this association as early as the 1980s, recent research has strongly suggests that negative affective states may significantly impacts on HRV, disease and poor health [63, 64]. Time and frequency domains measurements of HRV have been successfully used to index vagal activity and although there is debate regarding the reflection of Parasympathetic Nervous System (PNS) and/or Sympathetic Nervous System (SNS) influence of low frequency power, high frequency power has been shown to primarily reflect PNS activity with consensus amongst numerous studies is that lower index values of vagal function are associated with disease morbidity and mortality [65]. Three electrocardiogram (ECG) electrode pads will then be placed onto the participant’s upper left and upper right chest as well the lower left side of the torso. Participants will be asked to lay supine for 10 minutes during which the SphygmoCor system (AtCor Medical, Sydney, Australia) will capture data for HRV calculation. Data from both time (standard deviation of R to R intervals and root mean square successive differences) and frequency (high and low frequency spectral power and ratios) domain variables will be collected and analysed. This will provide indices that reflect the input to the heart from the two major branches of autonomic nervous system, the SNS and PNS.

Pulse Wave Velocity (PWV): Pulse Wave Velocity is generally accepted as the simplest, non-invasive, robust and reproducible method of determining arterial stiffness [66]. Research indicates it has independent predictive value for cardiovascular mortality and morbidity in a wide range of pathological and general populations [67–70]. Non-invasive PWV has been validated against invasive aortic PWV (correlation coefficient = 0.70) with suprasternal notch-femoral minus carotid-suprasternal notch distance showing the best agreement with invasive data [71]. While the participant is still supine, an ECG-femoral and carotid tonometric procedure will also be conducted. PWV procedure will be conducted in accordance to with recommendations outlined in the *Expert Consensus Document on Arterial Stiffness* [66]. Travel distance will be calculated by subtracting the carotid artery to suprasternal notch distance from the suprasternal notch to femoral artery. A single high fidelity applanation tonometer will then be placed over carotid and femoral arteries and data will be acquired for PWV. Travel time between the two arterial sites will be calculated as the difference between R-waves and the footpoints at their respective sites [71]. Footprint of the pressure wave at both sites will be automatically assessed using the intersecting-tangent method [71]. Calculations will be completed using SphygmoCor Cardiovascular Management Software (AtCor Medical, Sydney, Australia).

Carotid Intima Media Thickness (CIMT): Measurement of CIMT with B-mode ultrasound is a sensitive, non-invasive and reproducible technique for both identification and quantification of subclinical vascular disease and for evaluating CVD risk [72]. Imaging of the carotid artery to identify areas of increased thickness and non-occlusive atherosclerotic plaque which represents early stages of arterial injury and atherosclerosis has increasingly being used as a surrogate cardiovascular endpoint in a number of clinical trials [73]. Whilst there are numerous reviews and studies providing evidence that CIMT is strongly related to CVD incidents and that it is a well validated research tool , variations in methodology and sparse clinical data has prevented widespread adoption of this technique for routine assessment in clinical settings [74]. As a result, numerous guideline and consensus statements have been produced in an attempt to standardise CIMT procedures for research and clinical use. CIMT procedures in this study will be conducted in accordance with the *Mannheim Carotid Intima-Media Thickness Consensus (2004–2006)* [73]. The participant will be asked to lie in a supine position with their head tilted to the side. High resolution B-mode carotid ultrasonography will be performed using a 13Mhz linear-array transducer on a GE Vivid I ultrasound system (GE Medical, Sydney, Australia). Pre-processing configurations will be held constant during all examinations. The gain is to be adjusted so that the less dense arterial wall interface should be just visible and the image should clear show both near and far vessel walls. The Common Carotid Artery (CCA) will be insonated longitudinally and perpendicular to the vessel wall. Measurements are to be taken from the far wall of the CCA within 10 cm from the carotid bifurcation and of a minimal measurement length of 10 mm. IMT to be defined as the distance between the luminal-endothelial interface and the junction between the media and adventitia. Mean values of at least 10 measurements at systole are to be used for measurement. Image sets will be assessed for clarity and automatic edge tracking software known as EchoPak (GE Medical, Sydney, Australia) to be used offline to determine averaged mean and maximal IMT values and adventitia to adventitia diameter.

#### Cognitive function

Computerised Mental Performance Assessment System (COMPASS): The Computerised Mental Performance Assessment System (COMPASS) features a number of computerised cognitive tasks designed to test various aspects of mental function. The COMPASS test battery has been used in numerous cognitive studies and has been shown to be sensitive to changes in a number of cognitive domains [75–77]. The system employs a range of similarly validated cognitive tasks seen in computerised test suites such as the Cognitive Drug Research computerised assessment system however COMPASS is a purposely designed software application allowing for greater flexibility in choice of tasks and delivery of randomly generated parallel versions of standard cognitive assessment tasks [78]. Data from alternate forms of each task presented during testing sessions will be automatically captured by the COMPASS software.

#### Biochemistry markers

Low Density Lipoproteins (LDL), High Density Lipoproteins (HDL) and Triglycerides: Lipid levels, such as low High Density Lipoprotein (HDL) and high Low Density Lipoprotein (LDL) and triglyceride levels have been shown to be associated with increased risk of CVD [79]. Whilst the initiation of atherosclerosis has been long debated, mounting evidence suggests that LDLs interact with arterial walls allowing for prolonged exposure to local enzymes, both oxidative and non-, leading to modifications of lipoproteins and their constituents [80]. These modified lipoproteins have been suggested to play a role in inflammatory reactions that accelerate lesion development [81]. In contrast, research into HDLs highlight their functionality as being potentially highly vaso-protective in inhibiting endothelial apoptosis, maintain vaso-reactivity, contributing to endothelium repair and reducing adhesion molecule expression [82]. All of these actions may attenuate key processes of atherosclerotic plaque formation. In addition, population studies that provide evidence of associations between low HDLs and CVD risk are strong and consistent [79, 83]. Lipid profiles will be determined before and after the intervention at weeks 0 and 12. Participants will be sent to have blood samples taken and pathology testing conducted by a reputable pathology collection centre.

Glucose: Similarly to cholesterol, plasma glucose levels have been observed to be a continuous risk factor for CVD with diabetic patients exhibiting at least a 2-fold increase in risk [84]. However as traditional glucose cut off levels were chosen to identify diabetes mellitus and not increased CVD risk, people with elevated but below diabetic glucose concentrations still represent increased risk which has been highlighted in several large population studies [85–87]. Glucose profiles will be determined before and after the intervention at weeks 0 and 12. Participants will be sent to have blood samples taken and pathology testing conducted by a reputable pathology collection centre.

C-reactive protein (CRP): C-Reactive protein (CRP) was initially used as a non-specific biomarker for inflammatory processes however has become a powerful independent modifiable predictor of CVD in various clinical and non-clinical populations [88, 89]. The Centre for Disease Control and Prevention and the American Heart Association have issued a Class IIa recommendation that screening of CRP be a routine part of a patient’s global cardiovascular risk assessment and in primary prevention, CRP adds prognostic value to all levels of Framingham risk [90]. In addition to this, CRP was found to be a stronger predictor of CVD incidences that low density lipid levels [91]. CRP will be determined before and after the intervention at weeks 0 and 12. Participants will be sent to have blood samples taken and pathology testing conducted by a reputable pathology collection centre.

#### Participant diary

Participants will also be asked to complete a weekly diary documenting drastic changes in diet, commencement or cessation of negative behaviours such as alcohol consumption and smoking, number of hours spent exercising, changes in medication, significant positive or negative life events, adverse events, and compliance to yoga program. These behaviours will be monitored by open-ended questions to be completed weekly. This data will be presented for a descriptive synthesis only with compliance to yoga sessions and adverse events, coded into a binary yes/no format, to be considered for inclusion in analysis of covariance (ANCOVA) models as covariates.

#### Safety and adherence

In addition to data collected from participant diaries, each assessment visit will detail any information pertaining to adverse events and yoga session compliance will be monitored through class attendance. As stated previously, this data will be included for ANCOVA models as covariates.

### Statistical analysis

Analysis is to be performed using the R statistics package (version 3.1.1). All data will be inspected visually and statistically for normality (skewness and kurtosis between -1 and +1). Analysis of variance (ANOVA) and chi-squared tests of independence will be used to check for any differences between groups at study entry, any differences found here will be controlled for in that outcome analysis. Outcome analysis will take the form of ANCOVAs to detect changes between the experimental and control groups at post- treatment while controlling for baseline values and other possible confounders such as age, gender, time since cardiac incident, time since phase-2 CR completion and current exercise regimen or phase-3 CR participation. Multiple imputations will be used to impute missing data for participants with less than 10% missing data. An intention to treat analysis in which all missing data will be managed with multiple imputations will be conducted along with a separate per protocol analysis in which those with greater than 10% missing data or less than 60% yoga session attendance will be excluded. A p-value of less than 0.05 will be considered statistically significant.

## Discussion

Secondary prevention is an integral aspect regarding the comprehensive care of patients with CVD. Although it has been shown that CR is effective in positively modifying cardiac risk factors and reducing subsequent cardiac mortality, these benefits appear to plateau and gradually decrease upon completion of CR [92]. As noted by Gupta et al. [93] benefits achieved during CR programs are potentially sustainable at a one year follow up, however, minimal improvement across outcome measures was noted and significant regression was seen in important variables such as 6-minute walk distance, body mass index, diet scores, and smoking status. This is corroborated by evidence from Willich et al. [94] and Boesch et al. [95] who also reported worsening of lipid control at long-term follow points. According to adherence reports by Bittner et al. [96] and Ljubic et al. [97], approximately 50% of CR graduates do not maintain adherence to regular physical activity 5–6 months post-phase 2 CR and further highlights that this poor adherence may be associated with higher levels of psychosocial distress such as depression, anxiety, and stress. Given that numerous studies emphasis the capacity of yoga in positively modifying cardiovascular risk factors [35, 36, 38–45] in addition to psychosocial attenuation [28–34, 98], its application in post-cardiac rehabilitation may assist in sustaining and further supplementing the beneficial effects of phase 2 CR. As such, this study will aim to determine the effect of yoga practice on psychosocial, cardiovascular and cognitive function in post-phase 2 cardiac rehabilitation patients and is expected to provide insight into the short term impact of yoga on traditional and relatively novel cardiovascular risk factors and prognostic markers. In addition, these findings may also provide evidence supporting the incorporation of yoga into standardised CR programs as a practical adjunct to improve the management of psychosocial symptoms associated with CV events in addition to improving patients’ cognitive and cardiovascular functions.
